# The novel 2024 WHO *Neisseria gonorrhoeae* reference strains for global quality assurance of laboratory investigations and superseded WHO *N. gonorrhoeae* reference strains—phenotypic, genetic and reference genome characterization

**DOI:** 10.1093/jac/dkae176

**Published:** 2024-06-21

**Authors:** Magnus Unemo, Leonor Sánchez-Busó, Daniel Golparian, Susanne Jacobsson, Ken Shimuta, Pham Thi Lan, David W Eyre, Michelle Cole, Ismael Maatouk, Teodora Wi, Monica M Lahra

**Affiliations:** Department of Laboratory Medicine, Faculty of Medicine and Health, WHO Collaborating Centre for Gonorrhoea and Other STIs, National Reference Laboratory for STIs, Microbiology, Örebro University, Örebro, Sweden; Institute for Global Health, University College London (UCL), London, UK; Joint Research Unit ‘Infection and Public Health’, FISABIO-University of Valencia, Institute for Integrative Systems Biology (I2SysBio), Valencia, Spain; CIBERESP, ISCIII, Madrid, Spain; Department of Laboratory Medicine, Faculty of Medicine and Health, WHO Collaborating Centre for Gonorrhoea and Other STIs, National Reference Laboratory for STIs, Microbiology, Örebro University, Örebro, Sweden; Department of Laboratory Medicine, Faculty of Medicine and Health, WHO Collaborating Centre for Gonorrhoea and Other STIs, National Reference Laboratory for STIs, Microbiology, Örebro University, Örebro, Sweden; Department of Bacteriology I, National Institute of Infectious Diseases, Tokyo, Japan; Hanoi Medical University, National Hospital of Dermatology and Venereology, Hanoi, Vietnam; Big Data Institute, University of Oxford, Oxford, UK; Oxford University Hospitals NHS Foundation Trust, Oxford, UK; UK Health Security Agency (UKHSA), London, UK; Department of the Global HIV, Hepatitis and STI Programmes, WHO, Geneva, Switzerland; Department of the Global HIV, Hepatitis and STI Programmes, WHO, Geneva, Switzerland; WHO Collaborating Centre for Sexually Transmitted Infections and Antimicrobial Resistance, New South Wales Health Pathology, Microbiology, Randwick, NSW, Australia; Faculty of Medicine, The University of New South Wales, Sydney, Australia

## Abstract

**Objectives:**

MDR and XDR *Neisseria gonorrhoeae* strains remain major public health concerns internationally, and quality-assured global gonococcal antimicrobial resistance (AMR) surveillance is imperative. The WHO global Gonococcal Antimicrobial Surveillance Programme (GASP) and WHO Enhanced GASP (EGASP), including metadata and WGS, are expanding internationally. We present the phenotypic, genetic and reference genome characteristics of the 2024 WHO gonococcal reference strains (*n* = 15) for quality assurance worldwide. All superseded WHO gonococcal reference strains (*n* = 14) were identically characterized.

**Material and Methods:**

The 2024 WHO reference strains include 11 of the 2016 WHO reference strains, which were further characterized, and four novel strains. The superseded WHO reference strains include 11 WHO reference strains previously unpublished. All strains were characterized phenotypically and genomically (single-molecule PacBio or Oxford Nanopore and Illumina sequencing).

**Results:**

The 2024 WHO reference strains represent all available susceptible and resistant phenotypes and genotypes for antimicrobials currently and previously used (*n* = 22), or considered for future use (*n* = 3) in gonorrhoea treatment. The novel WHO strains include internationally spreading ceftriaxone resistance, ceftriaxone resistance due to new *penA* mutations, ceftriaxone plus high-level azithromycin resistance and azithromycin resistance due to mosaic MtrRCDE efflux pump. AMR, serogroup, prolyliminopeptidase, genetic AMR determinants, plasmid types, molecular epidemiological types and reference genome characteristics are presented for all strains.

**Conclusions:**

The 2024 WHO gonococcal reference strains are recommended for internal and external quality assurance in laboratory examinations, especially in the WHO GASP, EGASP and other GASPs, but also in phenotypic and molecular diagnostics, AMR prediction, pharmacodynamics, epidemiology, research and as complete reference genomes in WGS analysis.

## Introduction

Antimicrobial resistance (AMR) in *Neisseria gonorrhoeae* is compromising the treatment of gonorrhoea globally.^1–8^ Internationally, the extended-spectrum cephalosporin (ESC) ceftriaxone is the only remaining option for first-line empirical gonorrhoea therapy, i.e. given as a high-dose monotherapy or with azithromycin.^1,2,8–18^ However, gonococcal strains with resistance to ceftriaxone and especially azithromycin have been described globally.^2,5–10^ Furthermore, since 2015 international spread of the ceftriaxone-resistant MDR strain FC428 has been reported^5,10,19–22^ and since 2018 gonococcal XDR strains with ceftriaxone resistance combined with high-level azithromycin resistance have been described.^23–27^ Most of the currently identified ceftriaxone-resistant strains contain a mosaic *penA*-60.001 allele, which result in a mosaic penicillin-binding protein 2 (PBP2).^5,10,19–28^ The international spread of ceftriaxone-resistant MDR and XDR gonococcal strains and sporadic treatment failures with ceftriaxone (mainly of pharyngeal gonorrhoea) necessitate enhanced, quality-assured global gonococcal AMR surveillance.^1–3,6–8^

The WHO^3^ and ECDC^29,30^ have developed global and regional action plans, respectively, to control the transmission and impact of AMR gonococcal strains. One key component is to expand, improve and quality-assure the gonococcal AMR surveillance at local, national and global levels. The WHO global Gonococcal Antimicrobial Surveillance Programme (GASP) was relaunched in 2009 (www.who.int/initiatives/gonococcal-antimicrobial-surveillance-programme).^3,6–8^ Furthermore, the WHO Enhanced GASP (EGASP)^26,31–33^ is currently being expanded internationally (www.who.int/publications/i/item/9789240021341). WHO EGASP includes isolate AMR data linked to patient metadata and WGS, which is already implemented in some regional GASPs.^9,10^ To fulfil all the aims of WHO GASP and EGASP, valid, internationally comparable and quality-assured AMR data are imperative. This is enabled through the use of WHO reference strains.^34,35^ In 2016, the latest WHO gonococcal reference strain panel was published.^35^

Herein, the 2024 WHO gonococcal reference strain panel is presented and characterized in detail. This panel includes 11 of the 2016 WHO reference strains (*n* = 14),^35^ which were further characterized, and four novel WHO reference strains. These novel WHO strains represent highly relevant AMR phenotypes and/or genotypes that were not available for inclusion in the previous WHO reference strain panels.^34,35^ The novel WHO strains include the internationally spreading ceftriaxone-resistant, mosaic *penA*-60.001-containing FC428 strain (associated with several ceftriaxone treatment failures),^5,10,19–22^ one strain expressing ceftriaxone resistance due to a new *penA* mutation (associated with cefixime treatment failure),^36^ the first cultured strain with ceftriaxone resistance plus high-level azithromycin resistance (mosaic *penA*-60.001-containing and with 23S rRNA gene A2059G mutations, associated with ceftriaxone 1 g plus doxycycline treatment failure)^24^ and one internationally spreading azithromycin-resistant strain with a mosaic MtrRCDE efflux pump, i.e. with *Neisseria lactamica*-like mosaic 2 *mtrR* promoter and *mtrD* sequence.^10,37,38^ The 2024 WHO gonococcal reference strains were characterized in detail phenotypically {e.g. antibiograms [25 antimicrobials] and genetically [e.g. AMR determinants, multi-locus sequence typing (MLST),^39,40^*N. gonorrhoeae* multiantigen sequence typing (NG-MAST),^40,41^*N. gonorrhoeae* sequence typing for AMR (NG-STAR)^42^ and NG-STAR clonal complexes (CCs)^43^]}. Complete and characterized reference genomes are also described. These 2024 WHO gonococcal reference strains are recommended for internal and external quality assurance in all types of laboratory investigation, especially in the GASPs, e.g. the WHO global GASP,^6–8^ WHO EGASP^26,31–33^ and other international or national GASPs but also for phenotypic and molecular diagnostics, AMR prediction, pharmacodynamics, epidemiology, research and genomics. All superseded WHO gonococcal reference strains (*n* = 14), including 11 not previously published WHO reference strains that have been used internationally, were characterized similarly.

## Materials and methods

### Bacterial strains

The 2024 WHO gonococcal reference strains include 11 of the 2016 WHO gonococcal reference strains (*n* = 14)^35^ and four additional gonococcal strains. The novel strains are WHO H (Austria, 2011; ceftriaxone resistant due to a new *penA* mutation),^36^ WHO Q (UK, 2018; ceftriaxone resistant combined with high-level azithromycin resistance),^24^ WHO R (Japan, 2015; FC428, internationally spreading ceftriaxone resistant)^5,10,19–22^ and WHO S2 (Sweden, 2020; internationally spreading azithromycin-resistant strain due to a mosaic MtrRCDE efflux pump).^38^ Furthermore, all the superseded WHO reference strains (*n* = 14) were characterized. All strains were cultivated as described.^44^

### Detection of prolyliminopeptidase (PIP)

PIP^45^ production was detected using API NH (bioMérieux, Marcy l'Etoile, France) and genetically.

### Antimicrobial susceptibility testing

MIC values (mg/L) for 22 antimicrobials were determined using the Etest (bioMérieux) on GCRAP agar plates [3.6% Difco GC Medium Base agar (BD, Diagnostics, Sparks, MD, USA) with 1% haemoglobin (BD) and 1% IsoVitalex (BD)]. MICs of zoliflodacin,^46–54^ gepotidacin^55–57^ and lefamulin,^58,59^ were determined using agar dilution methodology. Clinical breakpoints or the epidemiological cut off (ECOFF, for azithromycin) from the EUCAST (v.14.0, https://www.eucast.org/clinical_breakpoints) were used, where available. For additional antimicrobials, only the consensus MIC values are presented. For all strains and antimicrobials, each determination was performed ≥3 times using new bacterial suspensions on separate batches of agar plates. β-lactamase production was detected using nitrocefin solution (Oxoid, Basingstoke, UK).

### Isolation of bacterial DNA

Genomic DNA for short-read and long-read sequencing was isolated using the QIAsymphony instrument (Qiagen, Hilden, Germany) and Nanobind CBB kit (PacBio, Menlo Park, CA, USA), respectively. Purified DNA was stored at 4°C before WGS.

### Whole-genome sequencing

Multiplexed PacBio Single-Molecule, Real-Time (SMRT) DNA genome sequencing was performed from post-shearing DNA fragment sizes (10.8–17 kb) using the Sequel System (PacBio), v.3.0 sequencing chemistry. The average length of the reads was 4120 bp and the sequencing depth averaged 335× (range 224–834×). Paired-end short-read sequencing was performed using Illumina NextSeq 550 with an average sequencing depth of 410× (range 198–597×).

Pacbio SMRT Tools v.7.0.1 indexed the long-read raw sequencing data in bam format using pbindex and convert it to fastq with bam2fastq. Genome assembly of these long reads were performed using both HGAP v.4.0^60^ and Canu v.1.9.^61^ Complete chromosomes were circularized starting on the *dnaA* using Circlator v1.5.5.^62^ Illumina short reads were mapped against the circularized chromosome with BWA-MEM v.0.7.17^63^ and the output filtered with samtools v.1.11^64^ to only keep proper-paired reads that map with a mapping quality of ≥25. These mappings were used to detect and fix base errors, small insertions/deletions (indels), local misassemblies and fill gaps in the initial long-read assembly using Pilon v.1.23.^65^ A minimum base and mapping qualities of 20 were required, and ≥25% of the reads mapping had to support a single nucleotide polymorphism (SNP) or indel. HGAP and Canu assemblies were compared using ACT v.18.1.^66^ To resolve discrepancies, we ran Trycycler v.0.4.1^67^ using the raw long-read data and both chromosome sequences from each strain. No changes were needed by Pilon on the Trycycler consensus assemblies. When required, a hybrid assembly approach with Unicycler v.0.4.9b^68^ was performed using the long- and short-read data. Depth of coverage was obtained by mapping to the final chromosome assemblies using pbmm2 (https://github.com/PacificBiosciences/pbmm2, based on minimap2^69^), and BWA-MEM, respectively, followed by the samtools depth command.

A short-read-only assembly was performed using SPAdes v.3.12^70^ with k-mer sizes of 21, 33, 55, 63, 77, 99, 111 and the *–*careful option to minimize mismatches and short indels. Both the long- and short-read assemblies were screened for the three known gonococcal plasmids, pCryptic, pBla and pConj,^35^ using blastn v.2.10.1+.^71^ The plasmids pCryptic, pBla and pConj were circularized starting on replication initiator protein, *repA* and TrfA gene using Circlator v.1.5.5, respectively.

Finalized circular chromosomes and plasmids were annotated using the National Center for Biotechnology Information (NCBI) Prokaryotic Genome Annotation Pipeline v.6.6,^72^ which also re-annotated the 2016 WHO gonococcal reference strains.^35^ Mapping of Illumina reads over the final assemblies was visually inspected using Artemis and sequencing depth across the genomes was obtained with samtools v.1.11. The core genome among the 29 strains was inferred using Panaroo v.1.2.6^73^ with default parameters and strict mode, polymorphic sites were obtained using SNP-sites^74^ and a maximum-likelihood tree was reconstructed from them using IQ-TREE v.2.0.3^75^ with automatic detection of the best substitution model^76^ (best-fit model TVM + F + ASC + R7) and 1000 ultrafast bootstrap replicates.^77^ Long-read sequencing data for WHO S2 was generated on a MinION Mk1C device (Oxford Nanopore Technologies) using a v.R10 flow cell (FLO-MIN114). The sequencing library was prepared without DNA fragmentation, and selection of long fragments (>3 kb) using duplex Nanopore chemistry (SQK-LSK114). Sequence data were deposited at the NCBI under BioProject PRJNA1067895.

Molecular sequence types (NG-MAST, NG-STAR and MLST)^39–42^ and AMR determinants were obtained from the *N. gonorrhoeae* scheme at Pathogenwatch.^10,78^ NG-STAR CCs were assigned using eBURST clustering on the NG-STAR ST database downloaded on 29 February 2024 (https://ngstar.canada.ca/).^43^ The number of copies of the 23S rRNA gene mutations, *pip* gene mutants and the presence of the *cppB* gene in the pCryptic plasmid were inspected manually in Artemis using the finalized assemblies. Individual genome characteristics were also obtained using Artemis. DNA uptake sequences (DUSs) were located in each chromosome using the EMBOSS application *fuzznuc.*^79^

## Results

### Phenotypic characterization

One (6.7%; WHO F) and 14 (93.3%) of the 2024 WHO reference strains belonged to serogroup PorB1a (WI) and PorB1b (WII/III), respectively (Table 1). One strain (6.7%; WHO U) was PIP-negative, and four (26.7%) strains (WHO M, O, R, and V) produced β-lactamase. The antimicrobial susceptibility testing results are described in Table 1. The strains represent all relevant, available resistant; susceptible, increased exposure; and susceptible phenotypes observed for most antimicrobials currently or previously recommended in national and international gonorrhoea treatment guidelines or antimicrobials in advanced clinical development for future treatment. These included strains with clinical resistance to ceftriaxone (*n* = 7), cefixime (*n* = 7), azithromycin (*n* = 5), spectinomycin (*n* = 1), ciprofloxacin (*n* = 10), penicillin G (*n* = 9) and tetracycline (*n* = 13), and high MICs of cefuroxime, cefepime, ceftaroline, ampicillin, temocillin, aztreonam, erythromycin, moxifloxacin, chloramphenicol, rifampicin and trimethoprim-sulfamethoxazole. No clinical strains with high MICs of ertapenem, gentamicin, kanamycin, fosfomycin, zoliflodacin, gepotidacin and lefamulin were available (Table 1).

**Table 1.** Serogroup, PIP production and antimicrobial susceptibility/resistance phenotypes displayed by the 2024 WHO *Neisseria gonorrhoeae* reference strains (*n* = 15), which are relevant for susceptibility testing of current, previous and novel therapeutic antimicrobials

Characteristics	WHO F^a^	WHO H	WHO K^a^	WHO L^a^	WHO M^a^	WHO O^a^	WHO P^a^	WHO Q	WHO R	WHO S2	WHO U^a^	WHO V^a^	WHO X^a^	WHO Y^a^	WHO Z^a^
NCTC number	13 477	15 081	13 479	13 480	13 481	13 483	13 484	14 208	15 082	15 083	13 817	13 818	13 820	13 821	13 822
Isolated (country, year)	Canada, 1991	Austria, 2011	Japan, 2003	Asia, 1996	Philippines, 1992	Canada, 1991	USA, Unknown	UK, 2018	Japan, 2015	Sweden, 2020	Sweden, 2011	Sweden, 2012	Japan, 2009	France, 2010	Australia, 2013
Serogroup	PorB1a	PorB1b	PorB1b	PorB1b	PorB1b	PorB1b	PorB1b	PorB1b	PorB1b	PorB1b	PorB1b	PorB1b	PorB1b	PorB1b	PorB1b
PIP production	Pos	Pos	Pos	Pos	Pos	Pos	Pos	Pos	Pos	Pos	−^b^	Pos	Pos	Pos	Pos
β−lactamase (PPNG)^c^	—	—	—	—	Pos^c^	Pos^c^	—	—	Pos^c^	—	—	Pos^c^	—	—	—
Ampicillin^d,e^	0.032	2	2	2	PPNG^c^ (8)	PPNG^c^(32)	0.064	2	PPNG^c^ (>256)	0.25	0.125	PPNG^c^ (>256)	2	0.5	2
Azithromycin^d^	S (0.25)	S (0.25)	S (0.5)	S (1)	S (0.5)	S (0.5)	R (4)	HLR (>256)	S (0.5)	R (2)	R (4)	HLR (>256)	S (0.5)	S (1)	S (1)
Aztreonam^d,e^	0.016	8	4	2	0.125	0.5	0.125	64	32	0.064	0.064	0.25	≥256	64	32
Cefepime^d,e^	<0.016	8	4	1	0.064	0.125	0.032	4	8	0.064	0.016	0.25	16	32	4
Cefixime^d^	S (<0.016)	R (0.5)	LLR (0.25)	S (0.125)	S (<0.016)	S (0.016)	S (<0.016)	HLR (2)	HLR (1)	S (<0.016)	S (<0.016)	S (<0.016)	HLR (4)	HLR (2)	HLR (2)
Ceftaroline^d,e^	0.004	0.5	0.125	0.5	0.064	0.25	0.064	0.5	0.5	0.064	0.016	0.25	2	4	0.5
Ceftriaxone^d^	S (<0.002)	LLR (0.25)	S (0.064)	LLR (0.25)	S (0.016)	S (0.032)	S (0.004)	R (0.5)	R (0.5)	S (0.008)	S (0.002)	S (0.064)	HLR (2)	HLR (1)	R (0.5)
Cefuroxime^d,e^	0.032	32	16	8	0.25	1	0.125	16	16	0.25	0.064	2	16	16	16
Chloramphenicol^d,e^	0.5	8	4	8	4	4	4	8	8	1	4	8	8	4	8
Ciprofloxacin^d^	S (0.004)	HLR (>32)	HLR (>32)	HLR (>32)	R (2)	S (0.008)	S (0.004)	HLR (>32)	HLR (>32)	S (0.032)	S (0.004)	HLR (>32)	HLR (>32)	HLR (>32)	HLR (>32)
Ertapenem^d,e^	<0.002	0.064	0.064	0.032	0.016	0.016	0.004	0.032	0.016	0.004	0.004	0.008	0.064	0.008	0.016
Erythromycin^d,e^	0.5	2	1	2	1	1	4	>256	2	8	>256	>256	2	2	4
Fosfomycin^d,e^	32	32	16	8	32	32	32	16	32	8	32	16	16	16	16
Gentamicin^d,e^	4	4	4	4	4	4	4	4	4	8	4	8	4	8	4
Gepotidacin^d,e^	0.125	0.5	0.5	4	2	0.5	0.5	1	0.25	1	0.25	0.25	0.5	0.5	0.5
Kanamycin^d,e^	16	16	16	32	16	16	16	16	16	16	8	16	16	16	8
Lefamulin^d,e^	0.125	0.5	0.5	0.5	0.5	0.5	2	0.5	0.5	1	0.5	2	0.5	0.5	0.5
Moxifloxacin^d,e^	0.004	4	8	>32	1	0.016	0.032	2	8	0.064	0.008	8	8	4	8
Penicillin G^d^	S (0.032)	R (2)	R (2)	R (2)	PPNG^c^ (≥32)	PPNG^c^ (>32)	I (0.25)	I (1)	PPNG^c^ (>32)	I (0.5)	I (0.125)	PPNG^c^ (>32)	R (4)	I (1)	R (2)
Rifampicin^d,e^	0.125	0.5	0.5	0.5	>32	0.25	>32	0.5	>32	0.5	0.25	0.5	0.5	0.5	0.5
Spectinomycin^d^	S (16)	S (8)	S (16)	S (16)	S (16)	R (>1024)	S (8)	S (8)	S (8)	S (16)	S (8)	S (16)	S (16)	S (16)	S (16)
Temocillin^d,e^	0.064	8	16	4	1	4	1	8	8	1	0.5	4	32	8	8
Tetracycline^d^	S (0.25)	R (4)	R (2)	R (2)	R (2)	R (2)	R (1)	TRNG (128)	R (4)	R (2)	R (1)	R (4)	R (2)	R (4)	R (4)
Trimethoprim-Sulfamethoxazole^d,e^	1	2	4	1	2	4	4	8	4	4	1	4	1	1	4
Zoliflodacin^d,e^	0.064	0.064	0.125	0.125	0.064	0.125	0.25	0.032	0.064	0.25	0.064	0.125	0.064	0.125	0.125

National Collection of Type Cultures (NCTC) susceptible; I, susceptible, increased exposure; R, resistant; PPNG, penicillinase-producing *N. gonorrhoeae*; LLR, low-level resistant; HLR, high-level resistant; TRNG, plasmid-mediated high-level tetracycline resistant *N. gonorrhoeae*.

^a^Include some previously published results.^35^ However, additional antimicrobials have been examined and some consensus MICs have slightly changed when additional MIC determinations using different MIC-determining methodologies have been performed.

^b^Do not produce the enzyme prolyliminopeptidase (PIP), which can result in doubtful and/or false-negative species identification of *N. gonorrhoeae* using biochemical or enzyme-substrate test. Global transmission of PIP-negative *N. gonorrhoeae* strains has been documented.^45^

^c^PPNG, penicillinase-producing *N. gonorrhoeae* (always considered resistant to all penicillins independent on identified MIC value, which might slightly vary).

^d^Resistance phenotypes based on MIC (mg/L) using Etest and agar dilution (zoliflodacin, gepotidacin, lefamulin), and clinical susceptibility/resistance breakpoints stated by the EUCAST (v.14.0; https://www.eucast.org/clinical_breakpoints), where available. The reported MIC values are mean MICs (rounded to whole MIC doubling dilution) and the acceptable range of the MICs for each antimicrobial and the different strains is ±1 MIC doubling dilution. Note: the consensus MICs shown should be used and interpreted with caution because these were derived using one Etest method only and, consequently, may slightly differ using other methods.

^e^No susceptibility/resistance breakpoints stated by the EUCAST (v.14.0; https://www.eucast.org/clinical_breakpoints).

The phenotypic characteristics of the superseded WHO reference strains (*n* = 14) are described in Table S1 (available as Supplementary data at *JAC* Online).

### Genetic characterization

WHO F harboured a wild-type *penA* allele, seven strains (WHO H, K, Q, R, X, Y, Z) contained six different mosaic *penA* alleles (main ESC resistance determinant)^1,2,9,10,19–28,42,80^ and seven strains displayed the D345 insertion in the β-lactam main target PBP2, which is frequently found in chromosomally mediated penicillin resistance (Tables 1 and 2).^1,2,42,80^ WHO Q and R contained the mosaic *penA*-60.001 allele that causes ceftriaxone resistance in most currently-spreading ceftriaxone-resistant strains.^5,10,19–28^ WHO H contained a PBP2 T534A mutation, which causes ceftriaxone and cefixime resistance.^36^ WHO L and Y harboured a PBP2 A501 V and A501P alteration, respectively, which can also increase the MICs of ESCs.^1,2,42,80,86,87^ WHO L, O and V contained PBP2 G542S or P551S, which also may increase the ESC MICs.^1,2,42,80,86,88^ None of the isolates carried any other known potential ceftriaxone-resistance mutations (e.g. *rpoB* P157L, G158 V or R201H or *rpoD* D92–95 deletion or E98K).^78,117^ Eleven strains contained a deletion of a single nucleotide (A; *n* = 9) or an A→C substitution (*n* = 2) in the 13 bp inverted repeat of the *mtrR* promoter sequence, resulting in an increased MtrCDE efflux of substrate antimicrobials, e.g. macrolides and β-lactam antimicrobials.^1,2,86,89–91^ Also WHO L has an over-expressed MtrCDE efflux pump, however, this is caused by its *mtr_120_* mutation, resulting in an additional promoter for *mtrCDE*.^92^ WHO S2 has a *N. lactamica*-like mosaic 2 *mtrR* promoter and *mtrD* sequence,^10,37,38,78^ while WHO P has a *N. meningitidis*-like mosaic 1 *mtrR* promoter and *mtrD* sequence.^10,78^ These mosaics increase the activity of the MtrCDE efflux pump and increase the MICs of antimicrobials such as macrolides.^10,78,93–97^ By contrast, a two base pair deletion in a GC dinucleotide repeat in *mtrC* decreases the MICs of antimicrobials, especially macrolides.^120^ However, this two base pair deletion was not found in any of the strains. Among the PorB1b strains (*n* = 14), all except WHO U displayed mutations in A102 [A102D (*n* = 10) and A102N (*n* = 3)] and 12 also a G101K alteration, which cause a decreased influx of target antimicrobials through the porin PorB1b.^1,2,86,99,100^ Twelve strains contained the L421P alteration in the second β-lactam target PBP1, which is found in high-level chromosomally mediated penicillin resistance.^101^ Of the β-lactamase-producing strains (*n* = 4), two (WHO M, O) contained African-type plasmid and two (WHO R, V) Asian-type plasmid, which harboured *bla_TEM-1_* (WHO M, O, V) or *bla_TEM-135_* (WHO R) resulting in high-level penicillin resistance (Tables 1 and 2).^1,86,111–113^ Ten strains contained GyrA S91F plus GyrA D95G (*n* = 4), D95N (*n* = 4) or D95A (*n* = 2) alterations, and nine of these strains additionally had 1–2 amino acid alterations in ParC D86, S87 or S88, which cause resistance to ciprofloxacin and other fluoroquinolones.^1,2,42,78,102^ One strain (WHO O) contained a C1192T spectinomycin target mutation in all four alleles of the 16S rRNA gene (spectinomycin MIC > 1024 mg/L^104^). One strain (WHO U) comprised the 23S rRNA C2611T gene mutation and two strains (WHO Q, V) harboured the 23S rRNA A2059G gene mutation that cause low- and high-level resistance to azithromycin, respectively.^1,2,42,106,107^ No azithromycin-resistance mutations were found in the *rplD* or *rplV* gene (encoding ribosomal protein L4 and L22, respectively)^78^ and none of the macrolide resistance-associated genes *mefA/E* (encoding Mef efflux pump),^118^*ereA* and *ereB* (encoding erythromycin esterase) or *ermA-C* and *ermF* (encoding RNA methylases that block macrolides from binding to the 23S subunit target)^119^ were identified. Three strains (WHO M, P, R) contained the H552N target mutation in RpoB (RNA polymerase subunit B), causing high-level rifampicin resistance.^109^ A *tet(M)-*carrying conjugative plasmid (Dutch type) causing high-level tetracycline resistance was detected in WHO Q (Tables 1 and 2).^86,114,115^ All strains except WHO F contained the V57M mutation in *rpsJ*, encoding ribosomal protein S10, contributing to chromosomally mediated tetracycline resistance.^86,108^ All strains except WHO F and WHO L contained the R228S mutation in the sulfonamide target dihydropteroate synthase (DHPS), encoded by *folP*, associated with sulfonamide resistance.^110^ Finally, no strain had any transcription-modulating mutations in the promoter sequence for the *macAB* operon (encoding the MacA-MacB efflux pump)^121^ or in the putative –35 promoter hexamer sequence (CTGACG) of the promoter sequence for the *norM* gene (encoding the NorM efflux pump) or in its ribosome binding site (TGAA).^122^

**Table 2.** Genetic characteristics of relevance for epidemiology, diagnostics and AMR in the 2024 WHO *Neisseria gonorrhoeae* reference strains (*n* = 15), which are relevant for susceptibility testing of current, previous and novel therapeutic antimicrobials

Characteristics	WHO F^a^	WHO H	WHO K^a^	WHO L^a^	WHO M^a^	WHO O^a^	WHO P^a^	WHO Q	WHO R	WHO S2	WHO U^a^	WHO V^a^	WHO X^a^	WHO Y^a^	WHO Z^a^
MLST sequence type (ST)^39,40^	ST10934	ST1901	ST7363	ST1590	ST7367	ST1902	ST8127	ST12039	ST1903	ST11422	ST7367	ST10314	ST7363	ST1901	ST7363
NG-MAST ST^40,41^	ST3303	ST1407	ST1424	ST1422	ST3304	ST495	ST3305	ST16848	ST3435	ST3935	ST2382	ST8927	ST4220	ST1407	ST4015
NG-STAR ST^42^	ST2	ST1582	ST4	ST5	ST6	ST8	ST9	ST996	ST233	ST193	ST224	ST225	ST226	ST16	ST227
NG-STAR clonal complex (CC)^43^	CC1401	CC90	CC348	CC1229	Ungroupable	CC26	CC63	CC73	CC199	CC63	CC2047	CC127	CC348	CC90	CC348
*porA* pseudogene mutant^81^	—	—	—	—	—	—	—	—	—	—	Yes	—	—	**—**	**—**
*cppB* gene^82–84^	—	Yes	Yes	Yes	Yes	Yes	Yes	Yes	Yes	Yes	Yes	Yes	Yes	Yes	Yes
*pip* gene mutant^45^	—	—	—	—	—	—	—	—	—	—	Yes	—	—	—	—
*penA* mosaic allele^2,4,9,10,19–28,42,80^	—	Yes	Yes	—	—	—	—	Yes	Yes	—	—	—	Yes	Yes	Yes
NG-STAR *penA* allele^42^	15.001	34.009	10.001	7.001	2.001	12.001	2.001	60.001	60.001	2.001	2.001	5.002	37.001	42.001	64.001
PBP2 A311^2,4,9,10,42,78,80,85,86^	—	—	—	—	—	—	—	A311V	A311V	—	—	—	A311V	—	A311V
PBP2 I312, G545^2,4,9,10,42,78,80,86,87^	—	I312M, G545S	I312M, G545S	—	—	—	—	I312M, G545S	I312M, G545S	—	—	—	I312M, G545S	I312M, G545S	I312M, G545S
PBP2 V316^2,4,9,10,42,78,80,85–87^	—	V316T	V316T	—	—	—	—	V316T	V316T	—	—	—	V316P	V316T	V316T
PBP2 D345 insertion^2,4,42,80^	—	—	—	Yes	Yes	Yes	Yes	—	—	Yes	Yes	Yes	—	—	—
PBP2 T483^2,4,85,86^	—	—	—	—	—	—	—	T483S	T483S	—	—	—	T483S	—	T483S
PBP2 A501^2,4,42,80,86,87^	—	—	—	A501V	—	—	—	—	—	—	—	—	—	A501P	—
PBP2 N512^2,4,85,86^	—	N512Y	N512Y	—	—	—	—	N512Y	N512Y	—	—	—	N512Y	N512Y	N512Y
PBP2 T534^36^	—	T534A	—	—	—	—	—	—	—	—	—	—	—	—	—
PBP2 G542^4,42,80,86,88^	—	—	—	G542S	—	—	—	—	—	—	—	G542S	—	—	—
PBP2 P551^4,42,80,86,88^	—	—	—	—	—	P551S	—	—	—	—	—	—	—	—	—
*mtrR* promoter; 13 bp inverted repeat^4,42,86,89–91^	—	A-del	A-del	—	A-del	A-del	A→C SNP	A-del	A-del	—	—	A-del	A-del	A-del	A→C SNP
*mtr_120_* ^92^	—	—	—	Yes	—	—	—	—	—	—	—	—	—	—	—
MtrR promoter mosaic^10,38,93–97^	—	—	—	—	—	—	Yes (99.4% Type 1)^10,79^	—	—	Yes (Type 2)^10,79^	—	—	—	—	—
MtrD mosaic^10,38,93–97^	—	—	—	—	—	—	Yes (Type 1)^10,79^	—	—	Yes (Type 2)^10,79^	—	—	—	—	—
MtrD R714, S821, K823^38,94–97^	—	—	—	—	—	—	—	—	—	S821A, K823E	—	—	—	—	—
MtrR A39, G45^4,89–91,98^	—	—	G45D	G45D	G45D	—	N/A^b^	G45D	—	—	—	—	—	—	—
*mtrR* coding region frame-shift mutation^4,35^	—	—	—	—	—	—	T-insert 60^b^	—	—	—	—	—	—	—	—
PorB1b G101^4,86,99,100^	N/A^c^	G101K	G101K	G101K	G101K	G101K	—	G101K	G101K	G101K	—	G101K	G101K	G101K	G101K
PorB1b A102^4,86,99,100^	N/A^c^	A102N	A102D	A102D	A102D	A102D	A102D	A102D	A102D	A102N	—	A102D	A102D	A102N	A102D
*ponA1*; PBP1 L421^101^	—	L421P	L421P	L421P	L421P	L421P	—	L421P	L421P	—	L421P	L421P	L421P	L421P	L421P
GyrA S91, D95^1,2,4,42,86,102^	—	S91F, D95G	S91F, D95N	S91F, D95N	S91F, D95G	—	—	S91F, D95A	S91F, D95A	—	—	S91F, D95G	S91F, D95N	S91F, D95G	S91F, D95N
GyrA A92^55,56^	—	—	—	—	—	—	—	—	—	—	—	—	—	—	—
GyrB D429, K450, S467^49–53^	—	—	—	—	—	—	—	—	—	—	—	—	—	—	—
ParC D86, S87 or S88^86,102^	—	S87R	S87R, S88P	D86N, S88P	—	—	—	S87R	S87R	—	—	S87R	S87R, S88P	S87R	S87R, S88P
ParE G410^103^	—	—	—	—	—	—	—	—	—	—	—	—	—	—	—
*16S rRNA* (C1192)^d,4,104^	—	—	—	—	—	C→T (4/)	—	—	—	—	—	—	—	—	—
RpsE T24^105^	—	—	—	—	—	—	—	—	—	—	—	—	—	—	—
*23S rRNA* (A2059, C2611)^d,1,2,4,42,106,107^	—	—	—	—	—	—	—	A→G (4/4)	—	—	C→T (4/4)	A→G (4/4)	—	—	—
*rpsJ* V57^86,108^	—	V57M	V57M	V57M	V57M	V57M	V57M	V57M	V57M	V57M	V57M	V57M	V57M	V57M	V57M
RpoB H552^109^	—	—	—	—	H552N	—	H552N	—	H552N	—	—	—	—	—	—
FolP R228^110^	—	R228S	R228S	—	R228S	R228S	R228S	R228S	R228S	R228S	R228S	R228S	R228S	R228S	R228S
ß-lactamase plasmid type^86,111–113^	—	—	—	—	African	African	—	—	Asian	—	—	Asian	—	—	—
*bla_TEM_* allele^112^	—	—	—	—	TEM-1	TEM-1	—	—	TEM-135	—	—	TEM-1	—	—	—
*tet(M)* plasmid type^86,114,115^	—	—	—	—	—	—	—	Dutch	—	—	—	—	—	—	—

Note: none of the 23S rRNA A2058,^116^*rplD*,^78^*rplV*,^78^*rpoB*,^78,117^*rpoD*,^78,117^*mef*,^118^*ereA*,^119^*ereB*,^119^*ermC*^119^ and *ermF*^119^ mutations associated with increased MICs of macrolides or cephalosporins were present.

ST, sequence type; PBP2, Penicillin-binding protein 2; rRNA, ribosomal RNA.

^a^Include some previously published results,^35^ however, many additional genes and mutations, and reference genomes have been characterized in the present paper.

^b^N/A, not applicable due to frame-shift mutation that causes a premature stop codon and truncated peptide.

^c^N/A, not applicable because these strains were of serogroup WI (PorB1a).

^d^ *Escherichia coli* numbering (A2045 and C2597, respectively, in *N. gonorrhoeae*). Number of the four alleles of the 23S rRNA gene with mutations is shown in parenthesis.

Regarding novel antimicrobials for gonorrhoea treatment, no strain contained any *gyrB* mutations associated with increased MICs of zoliflodacin (in GyrB D429 and K450) or predisposition for emergence of zoliflodacin resistance (GyrB S467N).^49–53^ Furthermore, no alterations in GyrA A92, i.e. one of the two targets for the new antimicrobial gepotidacin, was observed. However, one strain (WHO L) contained the ParC D86N alteration in the other gepotidacin target, i.e. which can predispose for emergence of gepotidacin resistance.^55,56^

Of importance for molecular (and/or phenotypic) detection of gonococci, *cppB*^81–83^ (WHO F), *pip*^45^ (WHO U) and *porA* pseudogene^84^ (WHO U) mutant strains were included. Finally, the strains represented 11, 14, 15 and 10 MLST STs, NG-MAST STs, NG-STAR STs and NG-STAR CCs (including one ungroupable strain), respectively (Table 2).

The genetic characteristics of the superseded WHO reference strains (*n* = 14) are described in Table S2.

### Reference genome characterization

The general characteristics of the reference genomes of the 2024 WHO gonococcal reference strains (*n* = 15) as well as the superseded WHO gonococcal reference strains (*n* = 14) are summarized in Table 3 and Table S3. The genome size ranged from 2 163 258 bp (WHO-β) to 2 308 468 bp (WHO A). The GC content, number of coding sequences (CDS) and average CDS size varied between 52.1%–52.7%, 1945–2125 and 836–856 bp. The number of core genes was 1791 and accessory genes varied from 248 to 402 (Table 3 and Table S3).

**Table 3.** General characteristics of the reference genomes of the 2024 WHO *Neisseria gonorrhoeae* reference strains (*n* = 15)

Characteristics	WHO F	WHO H	WHO K	WHO L	WHO M	WHO O	WHO P	WHO Q	WHO R	WHO S2	WHO U	WHO V	WHO X	WHO Y	WHO Z
Accession number	CP145052	CP145050-CP145051	CP145048-CP145049	CP145045-CP145047	CP145041-CP145044	CP145037-CP145040	CP145035-CP145036	CP145032-CP145034	CP145028-CP145031	CP145026-CP145027	CP145024-CP145025	CP145021-CP145023	CP145019-CP145020	CP145017-CP145018	CP145015-CP145016
Genome size (bp)	2 292 467	2 233 100	2 169 846	2 168 633	2 178 344	2 169 062	2 173 861	2 177 981	2 218 559	2 172 077	2 234 269	2 221 284	2 171 112	2 228 980	2 229 351
No. of CDS (without/with pseudogenes)	2125/2370	2036/2289	1952/2204	1955/2216	1982/2225	1971/2215	1961/2222	1963/2223	2020/2264	1964/2214	2036/2286	2039/2285	1961/2210	2028/2287	2033/2286
Coding density (%)	77.4	77.3	76.9	76.3	77.4	77.3	76.8	76.6	77.2	77.1	77.2	77.2	76.8	77.0	77.1
Average gene size (bp; without/with pseudogenes)	836/822	848/829	855/836	846/832	850/832	850/832	852/832	850/832	848/835	853/834	847/832	841/827	851/833	847/829	846/828
GC content (%)	52.1	52.3	52.6	52.6	52.6	52.6	52.6	52.6	52.4	52.6	52.4	52.4	52.6	52.4	52.4
5S rRNA	4														
16S rRNA	4														
23S rRNA	4														
tRNAs	55	55	56	55	55	55	55	55	55	55	55	55	56	55	56
ncRNAs	3														
tmRNAs	1														
No. genes in pangenome	2471														
No. core genes^a^	1791														
Accessory genes (%)	402 (18.3)	333 (15.7)	258 (12.6)	262 (12.8)	268 (13.0)	265 (12.9)	271 (13.1)	266 (12.9)	314 (14.9)	268 (13.0)	328 (15.5)	331 (15.6)	263 (12.8)	328 (15.5)	325 (15.4)
No. 10-mer DUS (12-mer DUS)^b^	1981 (1533)	1977 (1526)	1950 (1510)	1956 (1518)	1955 (1516)	1950 (1519)	1959 (1517)	1958 (1521)	1961 (1518)	1960 (1521)	1963 (1512)	1968 (1518)	1949 (1510)	1973 (1522)	1959 (1512)
Number of plasmids	0	1	1	2	3	3	1	2	3	1	1	2	1	1	1

bp, base pairs; CDS, coding sequence; GC, guanine-cytosine; rRNA, ribosomal RNA; tRNA, transfer RNA; ncRNA, non-coding RNA; tmRNA, transfer-messenger RNA.

^a^Present in 99%–100% of strains.

^b^Number of the 10-mer DUS sequence GCCGTCTGAA (no. of the 12-mer ATGCCGTCTGAA). Note: the 10-mer sequence is included in the 12-mer.

**Figure 1.**
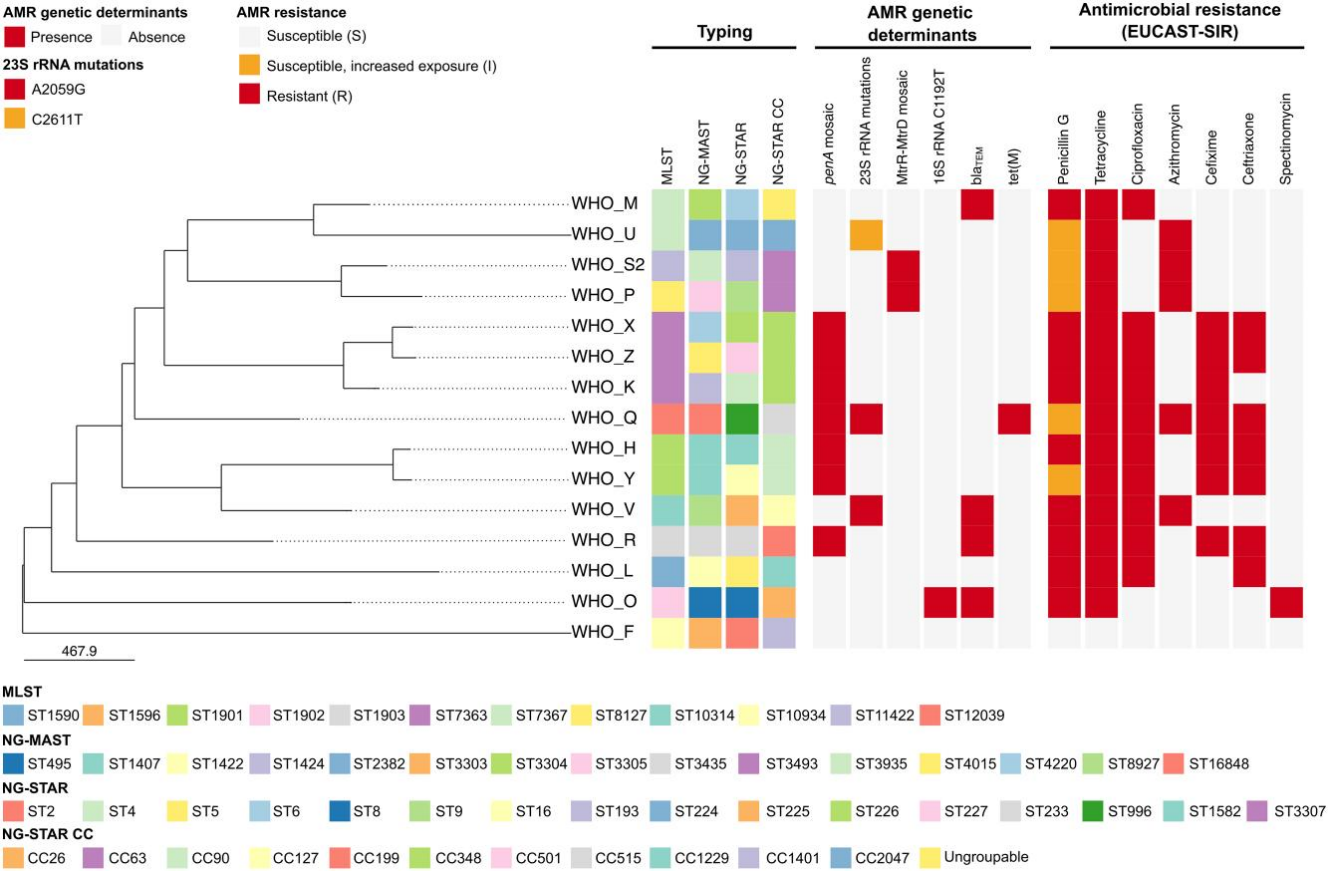
Phylogenomic tree of the 2024 WHO *Neisseria gonorrhoeae* reference core genomes (*n* = 15). Typing, key genetic determinants of AMR and phenotypic AMR patterns of the 2024 WHO gonococcal reference strains are shown alongside the tree. Only antimicrobials with EUCAST breakpoints (v.14.0, https://www.eucast.org/clinical_breakpoints) are displayed. This figure appears in colour in the online version of *JAC* and in black and white in the print version of *JAC*.

Figure 1 describes the phylogenomic relationship among all the 2024 WHO reference strain core genomes (*n* = 15, 1791 loci), including their molecular epidemiological types, key AMR determinants and phenotypic AMR patterns.

## Discussion

Herein, the 2024 WHO *N. gonorrhoeae* reference strains (and superseded WHO gonococcal reference strains) and their detailed phenotypic, genetic and reference genome characteristics are described. The utility of these strains includes internal and external quality assurance in all types of laboratory investigation, especially in the AMR testing (phenotypic and genetic) in GASPs, such as the WHO global GASP^6–8^ and WHO EGASP,^26,31–33^ but also for phenotypic (e.g. culture, species verification) and molecular (e.g. NAATs) diagnostics, AMR prediction, pharmacodynamics, epidemiology, research and genomics. The strains include all important global susceptible; susceptible, increased exposure; and resistant phenotypes and the ranges of resistances seen for most antimicrobials currently or previously recommended in national and international gonorrhoea treatment guidelines or antimicrobials in advanced clinical development for future treatment of gonorrhoea. However, the consensus MIC values (Table 1 and Table S1) were determined using one MIC-based method only (Etest). Accordingly, these MIC values may vary slightly using other MIC-based methods, however, the resistance phenotypes should be consistent. The 2024 WHO gonococcal reference strains are available through WHO sources and from the National Collection of Type Cultures (https://www.culturecollections.org.uk).

In many countries, NAATs have more or less replaced culture for gonococcal detection and, consequently, genetic detection of AMR determinants to predict resistance or susceptibility to antimicrobials has become increasingly important for AMR surveillance and, ideally, to also guide individually tailored treatment.^123–125^ The genetic AMR determinants that result in the different AMR phenotypes in the 2024 WHO gonococcal reference strains were characterized in detail and included most known gonococcal AMR determinants. Accordingly, the 2024 WHO reference strains can be used for internal and external quality assurance and quality controls of both conventional phenotypic AMR surveillance and surveillance using molecular AMR prediction. Molecular AMR methods can never entirely replace phenotypic culture-based AMR testing because they only detect known AMR determinants and new ones will continue to evolve. However, molecular prediction of AMR or susceptibility can supplement the phenotypic AMR surveillance, i.e. with varying sensitivity and specificity for different antimicrobials.^123–125^ The accuracy of the AMR prediction will also vary across geographic settings and time, due to the dynamics of the gonococcal population, regional variations in AMR and drug use, and evolution as well as importation of gonococcal strains in the settings. Finally, several challenges for direct testing of clinical, especially oropharyngeal, NAAT specimens and for accurate prediction of resistance to the currently recommended ceftriaxone and azithromycin remain.^123^ Nevertheless, WGS has revolutionized the molecular prediction of AMR or antimicrobial susceptibility, AMR surveillance and in general molecular epidemiological surveillance of *N. gonorrhoeae* strains nationally and internationally.^9,10,23,24,27,28,35,37,38,43,52,78,93,95,97,120,123^ However, to fully use the power of WGS joint analyses of quality-assured WGS, AMR and clinical and epidemiological data should be performed. This will substantially enhance the understanding of the spread, introduction, replacement, evolution and biofitness of AMR, and antimicrobial susceptible, clades/clones in risk groups nationally and internationally,^9,10^ which can inform gonorrhoea epidemiology, preventative measures, prediction of AMR or antimicrobial susceptibility, diagnostics and development of new antimicrobials and gonococcal vaccines. To support this development, we present the fully characterized and annotated chromosomes and plasmids of the 2024 WHO gonococcal reference strains, representing genomes that cover mainly the whole gonococcal species phylogeny (Figure S1), to enable quality assurance of *N. gonorrhoeae* WGS and its analysis. Ultimately, point-of-care genetic AMR methods, combined with gonococcal detection, should be used to guide individually tailored treatment of gonorrhoea, which can ensure rational use of antimicrobials (including sparing last-line antimicrobials) and affect the control of both gonorrhoea and gonococcal AMR.

The 2024 WHO *N. gonorrhoeae* reference strain panel includes 11 of the 2016 WHO reference strains (*n* = 14),^35^ which were further characterized, and four novel WHO reference strains. The four novel 2024 WHO strains (WHO H, Q, R and S2) represent phenotypes and/or genotypes that were not available when the 2016 WHO reference strains^35^ were published. Accordingly, WHO R is the first internationally spreading ceftriaxone-resistant strain FC428 (ceftriaxone caused by the mosaic *penA*-60.001 allele), associated with ceftriaxone treatment failures^5,10,19–22^; WHO Q is the first identified strain with ceftriaxone resistance (mosaic *penA*-60.001 allele) plus high-level azithromycin resistance (23S rRNA gene A2059G in all four alleles), associated with ceftriaxone 1 g plus doxycycline treatment failure^24^; WHO H is also expressing ceftriaxone resistance (mosaic *penA*-34.009, i.e. *penA*-34.001 plus the unique PBP2 T534A mutation), associated with cefixime treatment failure^36^ and WHO S2 is representing the main internationally spreading azithromycin-resistant clade (mosaic MtrRCDE efflux pump, i.e. with *Neisseria lactamica*-like mosaic 2 *mtrR* promoter and *mtrD* sequence^10,37,38,78^), which account for most of the mainly low-level azithromycin resistance in many countries.^10,37,38,78,93–95^ Furthermore, internationally spreading multidrug-resistant clones that have accounted for most of the ESC resistance globally such as MLST ST7363, ST1901 and ST1903, as well as NG-MAST ST1407, CC90 and CC199 are represented (Table 2).^4–6,9,10,19–22,38,43^ Notably, for the previously published WHO reference strains additional antimicrobial phenotypes and genotypes have been described and some consensus MICs have slightly changed when additional MIC determinations using different MIC-determining methodologies have been performed. Finally, all superseded WHO gonococcal reference strains (*n* = 14), including 11 not previously published WHO reference strains, were characterized in identical manners. It is important to provide quality-assured genetic and phenotypic characteristics for also these strains as they are still in use in some settings. Considering any historical data, the full characterization of the strains provides additional quality assurance to already published data. However, the use of the more relevant and updated 2024 WHO panel is strongly encouraged.

In conclusion, the 2024 WHO *N. gonorrhoeae* reference strains were extensively characterized both phenotypically and genetically, including characterizing the reference genomes, and are intended for internal and external quality assurance and quality control purposes in laboratory investigations. This is particularly in WHO GASP, WHO EGASP and other GASPs (to allow valid intra- and inter-laboratory comparisons of AMR data derived by different methods in various countries), but also in phenotypic (e.g. culture, species determination) and molecular diagnostics, genetic AMR detection, AMR prediction, pharmacodynamics, molecular epidemiology, research (including pre-clinical drug development) and as fully characterized, annotated and finished reference genomes in WGS analysis, transcriptomics, proteomics and other molecular technologies and data analysis. When additional resistant phenotypes and/or genotypes emerge, novel WHO gonococcal reference strains will be selected, characterized and added to the WHO gonococcal strain panel.

## Supplementary Material

dkae176_Supplementary_Data

## Supplementary data

Figure S1 and Tables S1 to S3 are available as Supplementary data at *JAC* Online.
