# Supplementary material for: The novel 2024 WHO Neisseria gonorrhoeae reference strains for global quality assurance of laboratory investigations and superseded WHO N. gonorrhoeae reference strains—phenotypic, genetic and reference genome characterization
Source: J Antimicrob Chemother. 2024 Jun 21;79(8):1885–99. doi: 10.1093/jac/dkae176 (PMC11290888; doi:10.1093/jac/dkae176)
Supplement: dkae176_Supplementary_Data [file dkae176_supplementary_data.doc]

**Table S1.** Serogroup, PIP production, and antimicrobial susceptibility/resistance phenotypes displayed by the superseded WHO *Neisseria gonorrhoeae* reference strains (n=14)

| **Characteristics** | **WHO A** | **WHO B** | **WHO C** | **WHO D** | **WHO E** | **WHO G*^a^*** | **WHO I** | **WHO J** | **WHO N*^a^*** | **WHO S** | **WHO T** | **WHO W*^a^*** | **WHO-α*^b^*** | **WHO-β*^b^*** |
| --- | --- | --- | --- | --- | --- | --- | --- | --- | --- | --- | --- | --- | --- | --- |
| Serogroup | PorB1b | PorB1b | PorB1b | PorB1b | PorB1a | PorB1a | PorB1b | PorB1b | PorB1a | PorB1b | PorB1b | PorB1b | PorB1b | PorB1b |
| PIP-production | Pos | Pos | Pos | Pos | Pos | -*^c^* | Pos | Pos | -*^c^* | Pos | Pos | Pos | Pos | Pos |
| β-lactamase (PPNG)*^d^* | - | - | - | - | Pos*^d^* | - | - | Pos*^d^* | Pos*^d^* | - |  | - | - | Pos*^d^* |
| Ampicillin*^e,f^* | 0.032 | 0.25 | 0.5 | 1 | PPNG*^d^* (8) | 0.25 | 1 | PPNG*^d^* (32) | PPNG*^d^* (32) | 0.032 | 1 | 2 | 2 | PPNG*^d^* (16) |
| Azithromycin*^e^* | S (0.064) | S (0.064) | S (0.5) | S (1) | S (0.25) | S (0.5) | S (0.25) | S (0.5) | S (0.25) | R (4) | S (0.5) | S (0.5) | S (1) | S (0.5) |
| Aztreonam*^e,f^* | 0.016 | 0.125 | 0.5 | 0.5 | 0.25 | 0.25 | 0.25 | 0.125 | 0.125 | 0.032 | 0.5 | 4 | 1 | 0.5 |
| Cefepime*^e,f^* | <0.016 | 0.032 | 0.25 | 0.25 | 0.032 | 0.064 | 0.125 | 0.125 | 0.032 | 0.032 | 0.25 | 4 | 1 | 0.125 |
| Cefixime*^e^* | S (<0.016) | S (<0.016) | S (0.032) | S (0.032) | S (<0.016) | S (<0.016) | S (0.032) | S (<0.016) | S (<0.016) | S (<0.016) | S (0.032) | LLR (0.25) | S (0.125) | S (0.032) |
| Ceftaroline*^e,f^* | 0.002 | 0.008 | 0.125 | 0.25 | 0.016 | 0.064 | 0.125 | 0.125 | 0.125 | 0.016 | 0.25 | 0.125 | 0.5 | 0.125 |
| Ceftriaxone*^e^* | S (<0.002) | S (0.004) | S (0.032) | S (0.064) | S (0.004) | S (0.008) | S (0.032) | S (0.016) | S (0.004) | S (<0.002) | S (0.032) | S (0.064) | S (0.125) | S (0.016) |
| Cefuroxime*^e,f^* | <0.016 | 0.032 | 1 | 2 | 0.125 | 0.25 | 1 | 0.5 | 0.25 | 0.125 | 1 | 16 | 4 | 1 |
| Chloramphenicol*^e,f^* | 0.5 | 0.5 | 1 | 8 | 2 | 2 | 8 | 4 | 4 | 0.5 | 8 | 8 | 8 | 1 |
| Ciprofloxacin*^e^* | S (0.004) | S (0.004) | S (0.004) | S (0.016) | S (0.004) | R (0.125) | R (0.5) | HLR (32) | R (4) | S (0.008) | S (0.016) | HLR (>32) | HLR (>32) | HLR (>32) |
| Ertapenem*^e,f^* | <0.002 | 0.004 | 0.016 | 0.016 | 0.008 | 0.008 | 0.008 | 0.004 | 0.004 | <0.002 | 0.016 | 0.064 | 0.016 | 0.008 |
| Erythromycin*^e,f^* | 0.125 | 0.064 | 0.5 | 4 | 0.5 | 1 | 2 | 2 | 1 | 4 | 2 | 2 | 2 | 2 |
| Fosfomycin*^e,f^* | 16 | 32 | 32 | 32 | 16 | 32 | 16 | 32 | 32 | 32 | 16 | 32 | 16 | 32 |
| Gentamicin*^e,f^* | 2 | 4 | 4 | 4 | 4 | 4 | 8 | 4 | 4 | 4 | 4 | 4 | 4 | 4 |
| Gepotidacin*^e,f^* | 0.125 | 0.125 | 0.5 | 0.5 | 0.125 | 1 | 1 | 2 | 0.25 | 0.5 | 0.5 | 0.5 | 0.5 | 1 |
| Kanamycin*^e,f^* | 16 | 16 | 16 | 16 | 16 | 16 | 32 | 32 | 16 | 16 | 16 | 16 | 16 | 16 |
| Lefamulin*^e,f^* | 0.064 | 0.016 | 0.25 | 0.5 | 0.125 | 0.25 | 0.5 | 0.5 | 0.25 | 0.25 | 0.25 | 0.5 | 0.5 | 0.25 |
| Moxifloxacin*^e,f^* | 0.008 | 0.008 | 0.016 | 0.032 | 0.008 | 0.064 | 0.25 | 4 | 2 | 0.032 | 0.032 | 8 | 8 | 2 |
| Penicillin G*^e^* | S (0.016) | I (0.125) | I (1) | R (4) | PPNG*^d^* (8) | I (0.5) | R (2) | PPNG*^d^* (>32) | PPNG*^d^* (>32) | S  (0.064) | R (2) | R (4) | I (1) | PPNG*^d^* (16) |
| Rifampicin*^e,f^* | 0.064 | 0.032 | 0.5 | 1 | 0.25 | 0.5 | 0.5 | >32 | >32 | 0.5 | >32 | 0.25 | 0.5 | >32 |
| Spectinomycin*^e^* | R (512) | S (16) | S (16) | S (16) | S (16) | S (16) | S (16) | S (8) | S (16) | S (16) | S (16) | S (16) | S (8) | S (16) |
| Temocillin*^e,f^* | <0.064 | 1 | 1 | 4 | 1 | 1 | 2 | 1 | 1 | <0.064 | 8 | 8 | 4 | 2 |
| Tetracycline*^e^* | S (0.25) | S (0.25) | R (1) | R (4) | S (0.5) | TRNG (32) | R (4) | R (4) | TRNG (16) | S (0.25) | R (2) | R (4) | R (2) | TRNG (128) |
| Trimethoprim-Sulfamethoxazole*^e,f^* | 0.5 | 1 | 1 | 8 | 0.5 | 8 | 2 | 8 | 4 | 4 | 2 | 2 | 8 | 4 |
| Zoliflodacin*^e,f^* | 0.032 | 0.008 | 0.125 | 0.125 | 0.064 | 0.125 | 0.125 | 0.125 | 0.125 | 0.125 | 0.125 | 0.125 | 0.125 | 0.064 |

S, susceptible; I, susceptible, increased exposure; R, resistant; PPNG, penicillinase-producing *N. gonorrhoeae;* LLR, low-level resistant; HLR, high-level resistant; TRNG, plasmid-mediated high-level tetracycline resistant *N. gonorrhoeae*.

*^a^*Include some previously published results.^35^ However, additional antimicrobials have been examined and some consensus MICs have slightly changed when additional MIC determinations using different MIC-determining methodologies have been performed.

*^b^*Reference strains available for further coverage of the gonococcal species phylogeny (n=2).

*^c^*Do not produce the enzyme prolyliminopeptidase (PIP), which can result in doubtful and/or false-negative species identification of *N. gonorrhoeae* using biochemical or enzyme-substrate test. Global transmission of PIP-negative *N. gonorrhoeae* strains has been documented.^45^

*^d^*PPNG, penicillinase producing *N. gonorrhoeae* (always considered resistant to all penicillins independent on identified MIC value, which might slightly vary).

*^e^*Resistance phenotypes based on MIC (mg/L) using Etest and agar dilution (zoliflodacin, gepotidacin, lefamulin), and clinical susceptibility/resistance breakpoints stated by the EUCAST (v14.0; https://www.eucast.org/clinical_breakpoints), where available. The reported MIC values are mean MICs (rounded to whole MIC doubling dilution) and the acceptable range of the MICs for each antimicrobial and the different strains is +/- one MIC doubling dilution. **Note:** the consensus MICs shown should be used and interpreted with caution because these were derived using one Etest method only and, consequently, may slightly differ using other methods.

*^f^*No susceptibility/resistance breakpoints stated by the EUCAST (v14.0; https://www.eucast.org/clinical_breakpoints).

**Table S2.** Genetic characteristics of relevance for epidemiology, diagnostics and antimicrobial resistance displayed by the superseded WHO *Neisseria gonorrhoeae* reference strains (n=14)

| **Characteristics** | **WHO A** | **WHO B** | **WHO C** | **WHO D** | **WHO E** | **WHO G*^a^*** | **WHO I** | **WHO J** | **WHO N*^a^*** | **WHO S** | **WHO T** | **WHO W*^a^*** | **WHO-α*^b^*** | **WHO-β*^b^*** |
| --- | --- | --- | --- | --- | --- | --- | --- | --- | --- | --- | --- | --- | --- | --- |
| MLST sequence type (ST)^39,40^ | ST10316 | ST15224 | ST10931 | ST1902 | ST10682 | ST1903 | ST1890 | ST1599 | ST1583 | ST1596 | ST1579 | ST7363 | ST7371 | ST1588 |
|  |  |  |  |  |  |  |  |  |  |  |  |  |  |  |
| NG-MAST ST^40,41^ | ST1752 | ST1488 | ST912 | ST917 | ST1699 | ST621 | ST270 | ST904 | ST556 | ST3493 | ST5 | ST835 | ST4787 | ST3611 |
| NG-STAR ST^42^ | ST1505 | ST3520 | ST3248 | ST243 | ST472 | ST3 | ST313 | ST312 | ST7 | ST5953 | ST139 | ST4 | ST501 | ST567 |
| NG-STAR clonal complex (CC)^43^ | CC352 | CC4959 | CC190 | Ungroupable | CC1115 | CC409 | CC334 | CC38 | CC208 | CC307 | CC442 | CC348 | CC501 | CC309 |
| *porA* pseudogene mutant^81^ | - | - | - | - | - | - | - | - | - | - | - | - | - | - |
| *cppB* gene^82-84^ | - | Yes | Yes | Yes | Yes | Yes | Yes | Yes | Yes | Yes | Yes | Yes | Yes | Yes |
| *pip* gene mutant^45^ | - | - | - | - | - | Yes | - | - | Yes | - | - | - | - | - |
| *penA* mosaic allele^2,4,9,10,19-28,42,80^ | - | - | - | - | - | - | - | - | - | - | - | Yes | - | - |
| NG-STAR *penA* allele^42^ | 100.008 | 1.002 | 156.001 | 12.001 | 22.001 | 2.001 | 12.001 | 2.002 | 2.001 | 15.001 | 9.001 | 10.001 | 18.001 | 19.001 |
| PBP2 A311^2,4,9,10,42,78,80,85,86^ | - | - | - | - | - | - | - | - | - | - | - | - | - | - |
| PBP2 I312, G545^2,4,9,10,42,78,80,86,87^ | - | - | - | - | - | - | - | - | - | - | - | I312M, G545S | - | - |
| PBP2 V316^2,4,9,10,42,78,80,85-87^ | - | - | - | - | - | - | - | - | - | - | - | V316T | - | - |
| PBP2 D345 insertion^2,4,42,80^ | - | Yes | Yes | Yes | Yes | Yes | Yes | Yes | Yes | - | Yes | - | Yes | Yes |
| PBP2 T483^2,4,85,86^ | - | - | - | - | - | - | - | - | - | - | - | - | - | - |
| PBP2 A501^2,4,42,80,86,87^ | - | - | - | - | - | - | - | - | - | - | - | - | A501T | - |
| PBP2 N512^2,4,85,86^ | - | - | - | - | - | - | - | - | - | - | - | N512Y | - | - |
| PBP2 T534^36^ | - | - | - | - | - | - | - | - | - | - | - | - | - | - |
| PBP2 G542^4,42,80,86,88^ | - | - | - | - | - | - | - | - | - | - | - | - | G542S | - |
| PBP2 P551^4,42,80,86,88^ | - | - | - | P551S | - | - | P551S | - | - | - | - | - | - | - |
| *mtrR* promoter; 13 bp inverted repeat^4,86,89-91^ | - | - | - | A-del | - | A-del | A-del | A-del | - | - | A-del | A-del | A-del | - |
| *mtr_120_*^92^ | - | - | - | - | - | - | - | - | - | - | - | - | - | - |
| MtrR promoter mosaic^10,38,93-97^ | - | - | - | - | - | - | - | - | - | Yes  (97.6% Type 2)^10,79^ | - | - | - | - |
| MtrD mosaic^10,38,93-97^ | - | - | - | - | - | - | - | - | - | Yes  (92.8% Type 3)^10,79^ | - | - | - | - |
| MtrD R714, S821, K823^38,94-97^ | - | - | - | - | - | - | - | - | - | S821A, K823E | - | - | - | - |
| MtrR A39, G45^4,89-91,98^ | A39T | - | G45D | G45D | A39T | - | - | G45D | A39T | - | - | G45D | A39T | A39T |
| *mtrR* coding region frame shift mutation^4,35^ | - | - | - | - | - | - | - | - | A-del 158 | - | - | - | - | CC-ins 382 |
| PorB1b G101^4,86,99,100^ | - | - | G101D | G101K | N/A*^c^* | N/A*^c^* | G101K | G101K | N/A*^c^* | - | G101K | G101K | G101K | G101K |
| PorB1b A102^4,86,99,100^ | - | A102S | - | A102N | N/A*^c^* | N/A*^c^* | A102D | A102D | N/A*^c^* | - | A102D | A102D | A102D | A102G |
| *ponA1*; PBP1 L421^101^ | - | - | - | L421P | L421P | L421P | L421P | L421P | L421P | - | L421P | L421P | L421P | L421P |
| GyrA S91, D95^1,2,4,42,86,102^ | - | - | - | - | - | S91F | S91F | S91F, D95G | S91F, D95G | - | - | S91F, D95N | S91F, D95N | S91F, D95A |
| GyrA A92^55,56^ | - | - | - | - | - | - | - | - | - | - | - | - | - | - |
| GyrB D429, K450, S467^49-53^ | - | - | - | - | - | - | - | - | - | - | - | - | - | - |
| ParC D86, S87 or S88^86,102^ | - | - | - | - | - | - | - | D86N | S87I | - | - | S87R, S88P | S87I | S87N |
| ParE G410^103^ | - | - | - | - | - | G410V | - | - | G410V | - | - | -- |  | - |
| *16S rRNA* (C1192)*^d^,*^4,104^ | - | - | - | - | - | - | - | - | - | - | - | - | - | - |
| RpsE T24^105^ | T24P | - | - | - | - | - | - | - | - | - | - | - | - | - |
| *23S rRNA* (A2059, C2611)*^d^,*^1,2,4,42,106,107^ | - | - | - | - | - | - | - | - | - | C→T (1/4) | - | - | - | - |
| *rpsJ* V57^86,108^ | - | - | V57M | V57M | V57M | V57M | V57M | V57M | V57M | - | V57M | V57M | V57M | V57M |
| RpoB H552^109^ | - | - | - | - | - | - | - | - | H552N | - | H552N | - | - | H552N |
| FolP R228^110^ | - | - | - | R228S | - | R228S | R228S | R228S | R228S | R228S | R228S | R228S | R228S | R228S |
| ß-lactamase plasmid type^86,111-113^ | - | - | - | - | African | - | - | Asian | Asian | - | - | - | - | African |
| *bla_TEM_* allele^112^ | - | - | - | - | TEM-1 | - | - | TEM-1 | TEM-1 | - | - | - | - | TEM-1 |
| *tet(M)* plasmid type^86,114,115^ | - | - | - | - | - | Dutch | - | - | Dutch | - | - | - | - | American |

ST, sequence type; NG-MAST, *N. gonorrhoeae* multiantigen sequence typing; NG-STAR, *N. gonorrhoeae* sequence typing for antimicrobial resistance; CC, clonal complex; PBP2, Penicillin-binding protein 2; rRNA, ribosomal RNA.

*^a^*Include some previously published results,^35^ however, many additional genes and mutations, and reference genomes have been characterised in the present paper.

*^b^*Reference strains available for further coverage of the gonococcal species phylogeny (n=2), see Figure S1.

*^c^*N/A, not applicable because these strains were of serogroup WI (PorB1a).

*^d^Escherichia coli* numbering numbering (A2045 and C2597, respectively, in *N. gonorrhoeae*). Mutations found in all four alleles of the gene.

NOTE: None of the 23S rRNA A2058,^116^ *rplD*,^78^ *rplV*,^78^ *rpoB*,^117^ *rpoD*,^117^ *mef*,^118^ *ereA*,^119^ *ereB*,^119^ *ermC*,^119^ and *ermF*^119^ mutations associated with increased MICs of macrolides or cephalosporins were present.

**Table S3.** General characteristics of the reference genomes of the superseded WHO *Neisseria gonorrhoeae* reference strains (n=14)

| **Characteristics** | **WHO A** | **WHO B** | **WHO C** | **WHO D** | **WHO E** | **WHO G** | **WHO I** | **WHO J** | **WHO N** | **WHO S** | **WHO T** | **WHO W** | **WHO**-α | **WHO-β** |  |
| --- | --- | --- | --- | --- | --- | --- | --- | --- | --- | --- | --- | --- | --- | --- | --- |
| Accession number | CP145090 | CP145088-CP145089 | CP145086-CP145087 | CP145083-CP145085 | CP145080-CP145082 | CP145077-CP145079 | CP145074-CP145076 | CP145071-CP145073 | CP145067-CP145070 | CP145064-CP145066 | CP145062-CP145063 | CP145059-CP145061 | CP145057-CP145058 | CP145053-CP145056 |  |
| Genome size (bp) | 2,308,468 | 2,219,659 | 2,167,377 | 2,178,180 | 2,213,585 | 2,167,361 | 2,171,276 | 2,168,993 | 2,172,826 | 2,221,865 | 2,247,111 | 2,222,386 | 2,235,802 | 2,163,258 |  |
| No. of CDS (without/with pseudogenes) | 2,106/2,369 | 2,027/2,262 | 1,964/2,212 | 1,986/2,234 | 2,008/2,259 | 1,959/2,206 | 1,959/2,211 | 1,951/2,205 | 1,965/2,217 | 2,023/2,268 | 2,042/2,289 | 2,037/2,282 | 2,034/2,284 | 1,945/2,200 |  |
| Coding density (%) | 76.9 | 77.6 | 76.8 | 77.0 | 76.9 | 77.0 | 76.9 | 76.6 | 77.0 | 77.1 | 77.7 | 77.6 | 77.0 | 77.0 |  |
| Average gene size (bp; without/with pseudogenes) | 843/829 | 850/836 | 848/832 | 844/829 | 848/834 | 852/834 | 852/834 | 852/836 | 852/831 | 846/833 | 855/836 | 847/829 | 847/833 | 856/836 |  |
| GC content (%) | 52.1 | 52.4 | 52.6 | 52.6 | 52.5 | 52.6 | 52.6 | 52.7 | 52.6 | 52.4 | 52.5 | 52.4 | 52.4 | 52.6 |  |
| 5S rRNA | 4 | | | | | | | | | | | | | | |
| 16S rRNA | 4 | | | | | | | | | | | | | | |
| 23S rRNA | 4 | | | | | | | | | | | | | | |
| tRNAs | 54 | 55 | 55 | 55 | 55 | 55 | 55 | 54 | 55 | 55 | 55 | 55 | 55 | 55 |  |
| ncRNAs | 3 | | | | | | | | | | | | | | |
| tmRNAs | 1 | | | | | | | | | | | | | | |
| No. genes in pangenome | 2,471 | | | | | | | | | | | | | | |
| No. core genes*^a^* | 1,791 | | | | | | | | | | | | | | |
| Accessory genes (%) | 402 (18.3) | 313 (14.9) | 253 (12.4) | 276 (13.4) | 298 (14.3) | 253 (12.4) | 257 (12.5) | 251 (12.3) | 263 (12.8) | 306 (14.6) | 337 (15.8) | 318 (15.1) | 329 (15.5) | 248 (12.2) |  |
| No. 10-mer DUS (12-mer DUS)*^b^* | 1,981 (1,534) | 1,962 (1,516) | 1,968 (1,526) | 1,951 (1,516) | 1,957 (1,515) | 1,947 (1,510) | 1,960 (1,517) | 1,951 (1,518) | 1,951 (1,513) | 1,970 (1,524) | 1,977 (1,537) | 1,954 (1,509) | 1,961 (1,512) | 1,938 (1,507) |  |
| Number of plasmids | 0 | 1 | 1 | 2 | 2 | 2 | 2 | 2 | 3 | 2 | 1 | 2 | 1 | 3 |  |

bp, base pairs; CDS, coding sequence; GC, guanine-cytosine; rRNA, ribosomal RNA; tRNA, transfer RNA; ncRNA, non-coding RNA; tmRNA, transfer-messenger RNA; DUS, DNA uptake sequence.

*^a^* Present in 99-100% of strains.

*^b^* Number of the 10-mer DUS sequence GCCGTCTGAA (no. of the 12-mer ATGCCGTCTGAA). **Note:** The 10-mer sequence is included in the 12-mer.


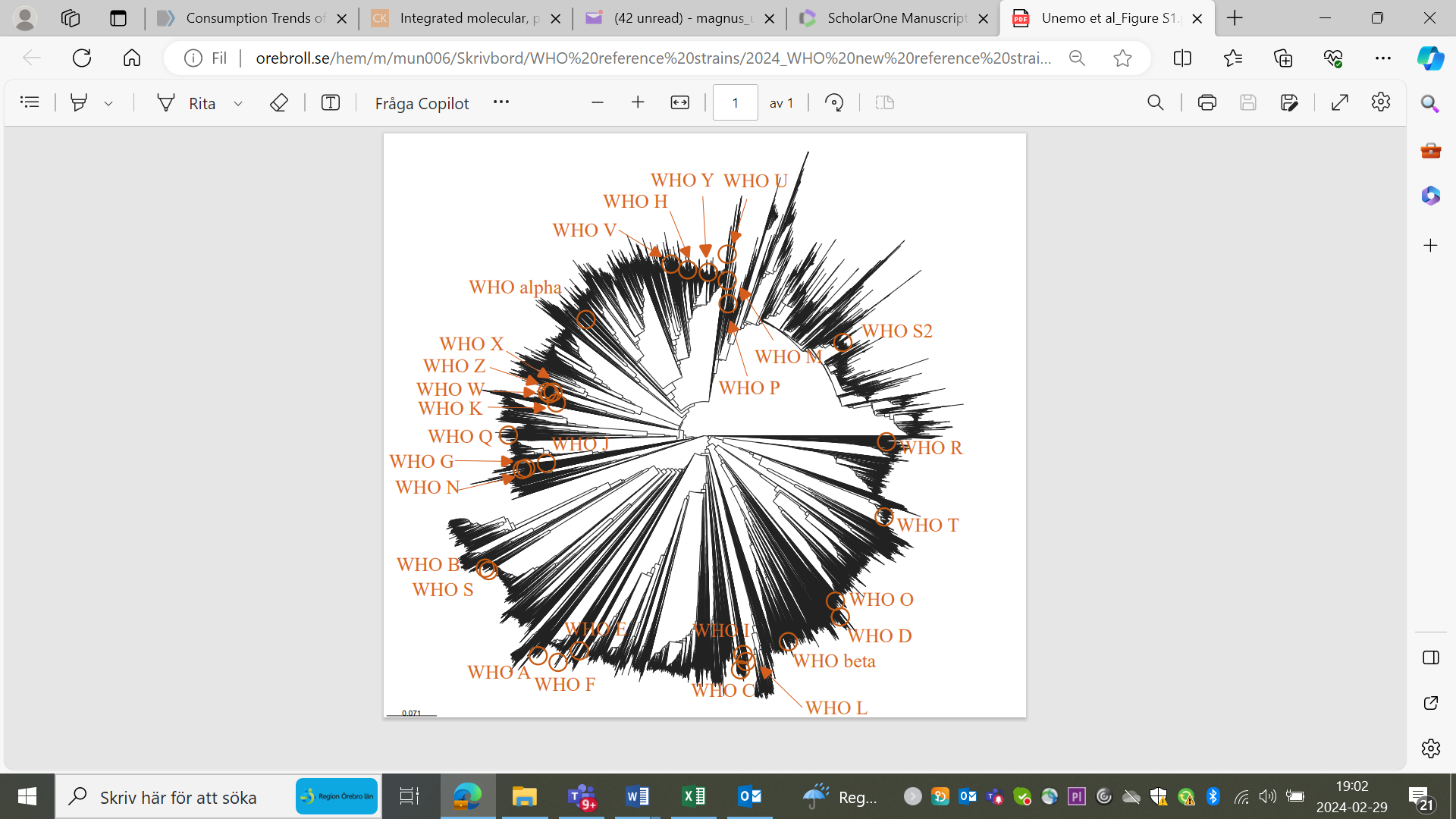


**Figure S1.** Phylogenomic analysis based on core genome MLST of 2062 gene alleles of Neisseria gonorrhoeae genomes (n = 45414) obtained from the European Nucleotide Archive (short-read archive). The locations of all the 2024 WHO *N. gonorrhoeae* reference strains and the superseded WHO *N. gonorrhoeae* reference strains are illustrated.
